# Planar coil-based contact-mode magnetic stimulation: synaptic responses in hippocampal slices and thermal considerations

**DOI:** 10.1038/s41598-018-31536-w

**Published:** 2018-09-07

**Authors:** Hee-Jin Park, Heekyung Kang, Jihoon Jo, Euiheon Chung, Sohee Kim

**Affiliations:** 1 School of Mechanical Engineering, Gwangju Institute of Science and Technology (GIST), Gwangju, Republic of Korea; 2 Department of Biomedical Science and Neurology, Chonnam National University Medical School, Gwangju, Republic of Korea; 3 Biomedical Research Institute, Chonnam National University Hospital, Gwangju, Republic of Korea; 4 Department of Biomedical Science and Engineering, Institute of Integrated Technology, Gwangju Institute of Science and Technology (GIST), Gwangju, Republic of Korea; 5 Department of Robotics Engineering, Daegu Gyeongbuk Institute of Science and Technology (DGIST), Daegu, Republic of Korea

## Abstract

Implantable magnetic stimulation is an emerging type of neuromodulation using coils that are small enough to be implanted in the brain. A major advantage of this method is that stimulation performance could be sustained even though the coil is encapsulated by gliosis due to foreign body reactions. Magnetic fields can induce indirect electric fields and currents in neurons. Compared to transcranial magnetic stimulation, the coil size used in implantable magnetic stimulation can be greatly reduced. However, the size reduction is accompanied by an increase in coil resistance. Hence, the coil could potentially damage neurons from the excess heat generated. Therefore, it is necessary to study the stimulation performance and possible thermal damage by implantable magnetic stimulation. Here, we devised contact-mode magnetic stimulation (CMS), wherein magnetic stimulation was applied to hippocampal slices through a customized planar-type coil underneath the slice in the contact mode. With acute hippocampal slices, we investigated the synaptic responses to examine the field excitatory postsynaptic responses of CMS and the temperature rise during CMS. A long-lasting synaptic depression was exhibited in the CA1 stratum radiatum after CMS, while the temperature remained in a safe range so as not to seriously affect the neural responses.

## Introduction

Implantable magnetic stimulation (IMS) has recently been suggested as a new form of brain stimulation^1–3^. Unlike transcranial magnetic stimulation (TMS), which is widely used as one of the non-invasive brain stimulation methods for diagnostic and therapeutic purposes^4^, IMS uses small implantable coils to deliver neuronal stimulation via electromagnetic induction. IMS has certain advantages over conventional implantable electrical stimulation. Implantable electrical stimulation methods apply currents directly to neurons, and neural tissues need to be in contact with conductive materials. However, conductive electrodes can be encapsulated by glial fibrosis due to inflammatory responses in the body over time^5–9^. These inflammatory reactions make it difficult to stimulate neurons because such encapsulation layer insulates the electrode from nearby neurons^10–12^, possibly hindering electric charge diffusion from the electrode, increasing electrical impedance of the electrode^13,14^, and increasing the distance between the stimulating surface and nearby neuronal cell bodies, ranging from a few tens of micrometers up to several hundreds of micrometers^13,15,16^. Increasing the stimulation intensity to circumvent such fibrosis encapsulation problems leads to the increased risks of tissue damage as well as electrode damage^17^. On the other hand, the IMS intensity would be sustained, even when the implanted coil may be covered with macrophages and giant cells by foreign body reactions. The magnetic fields in IMS induce electric fields, which evoke excitatory or inhibitory responses in neurons. In addition, a significantly reduced coil size enables more localized stimulation than conventional TMS^18,19^.

With these advantages, the feasibility of IMS has been studied using *in vivo*^20^ and *in vitro* models^18^. Based on those models, an invasive IMS device has been developed^2^. Despite these recent studies, there has been little discussion about the safety issues involved in implantable magnetic stimulation. These must be verified for any implantable devices that are to be used for diagnosis or treatment purposes in the body. One of the side effects of TMS is the risk of burns^21^. IMS also creates heat problems when coils are miniaturized^22^. Excess heat generation within the coils by high currents, required to evoke neural responses, may affect normal cellular physiology when the coils are implanted within the body.

For IMS, many parameters in terms of coil configurations and stimulation pulses need to be carefully adjusted. Recently, we conducted a simulation study to find the proper range of parameters that can satisfy both the safety limit and stimulation requirements^23^. Heat transfer and electromagnetic field distributions were investigated depending on the configuration of the coil and stimulation protocols. Among the various combinations of parameters, one feasible parameter set required a 10 mm outer diameter, 1 mm inner diameter, 30 μm line width and spacing, and 75 turns for the coil, and 40 V stimulating voltage. In repetitive transcranial magnetic stimulation (rTMS), quadripulse stimulation (QPS) was found to satisfy both the activation of neural cells and the temperature stayed under 40 °C^24–26^.

In magnetic stimulation studies targeting the hippocampus, many studies employed high-frequency stimulation (HFS), for instance, to promote neurogenesis in a transgenic mouse model of Alzheimer’s disease^27^ and to investigate long-term potentiation (LTP) effects in acute hippocampal slices^28,29^ and organotypic cultured slices^30^. Few results have been related to the synaptic responses using low-frequency magnetic stimulation. Lenz and colleagues recently found that 10 Hz rTMS elicited plasticity of inhibitory synapses in organotypic cultured slices^31^. Moreover, Wieraszko confirmed that seizure or amplification of population spikes was observed using pulsed magnetic stimulation protocols with very low repetition rates^32^. Based on recent experimental evidences showing that the stimulation frequency is a crucial parameter for altering inhibitory or excitatory synaptic responses, QPS is an interesting stimulus pattern that contains both low and high frequencies.

In the present study, we devised a form of IMS called ‘contact-mode magnetic stimulation (CMS)’. The tissue and the coil were as close as possible to each other in order to facilitate future implantation of planar coil-based CMS systems in the human body. In previous studies, TMS coils or micro coils were placed in a non-contact manner to stimulate cells or tissues^18,20,28^, while our experimental setup used a coil that was placed underneath the hippocampal slices. Thus, the heat was directly transferred by conduction to the hippocampal slice during magnetic stimulation. We examined the effects of QPS-patterned CMS on synaptic activities in hippocampal slices by electrophysiological recording and subsequently measured the temperature changes around the coil. This allowed us to determine whether the heating by the coil would be in a safe range without cell damage.

## Results

### Planar coils for contact-mode magnetic stimulation

**Figure 1:**
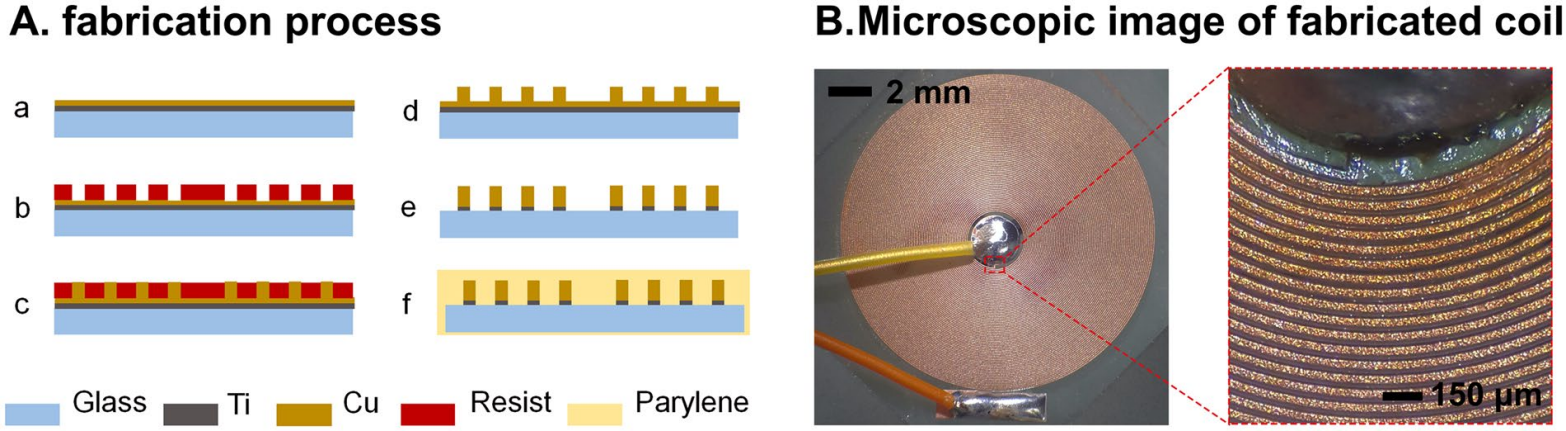
Planar coil-based implantable magnetic stimulator: (**A**) Schematic of the coil fabrication processes: (a) sputtering of the Ti/Cu seed layer for electroplating, (b) lithography using a photoresist to pattern conductive coil lines, (c) copper electroplating, (d) photoresist removal, (e) chemical etching of the Ti/Cu seed layer, and (f) parylene-C passivation. (**B**) Microscopic images of the fabricated coil. Its electrical properties are as follows: inductance of 26.3 ± 1.5 μH, capacitance of 1.31 ± 0.2 pF and resistance of 31.02 ± 1.7 Ω. The geometrical parameters of the coil were line width and spacing of 30 μm, outer diameter of 11 mm, inner diameter of 2 mm, and 75 turns.

A small-sized implantable planar spiral coil was manufactured by using microfabrication processes. The coil design was based on our previous simulation results^1^. The overall fabrication processes are shown in Fig. 1A. The coil with micro conductor lines was produced by photolithography, electroplating, sputtering, and various chemical etching techniques (for the more detailed procedure, see the Methods section). The geometrical parameters of the coil were line width and spacing of 30 μm, an inner diameter of 2 mm, an outer diameter of 11 mm, and 75 turns (Fig. 1B). The fabricated coils (n = 8) resulted in inductance of 26.3 ± 1.5 μH (mean ± standard deviation), the capacitance of 1.31 ± 0.2 pF and resistance of 31.02 ± 1.7 Ω.

### Synaptic responses by contact-mode magnetic stimulation

**Figure 2:**
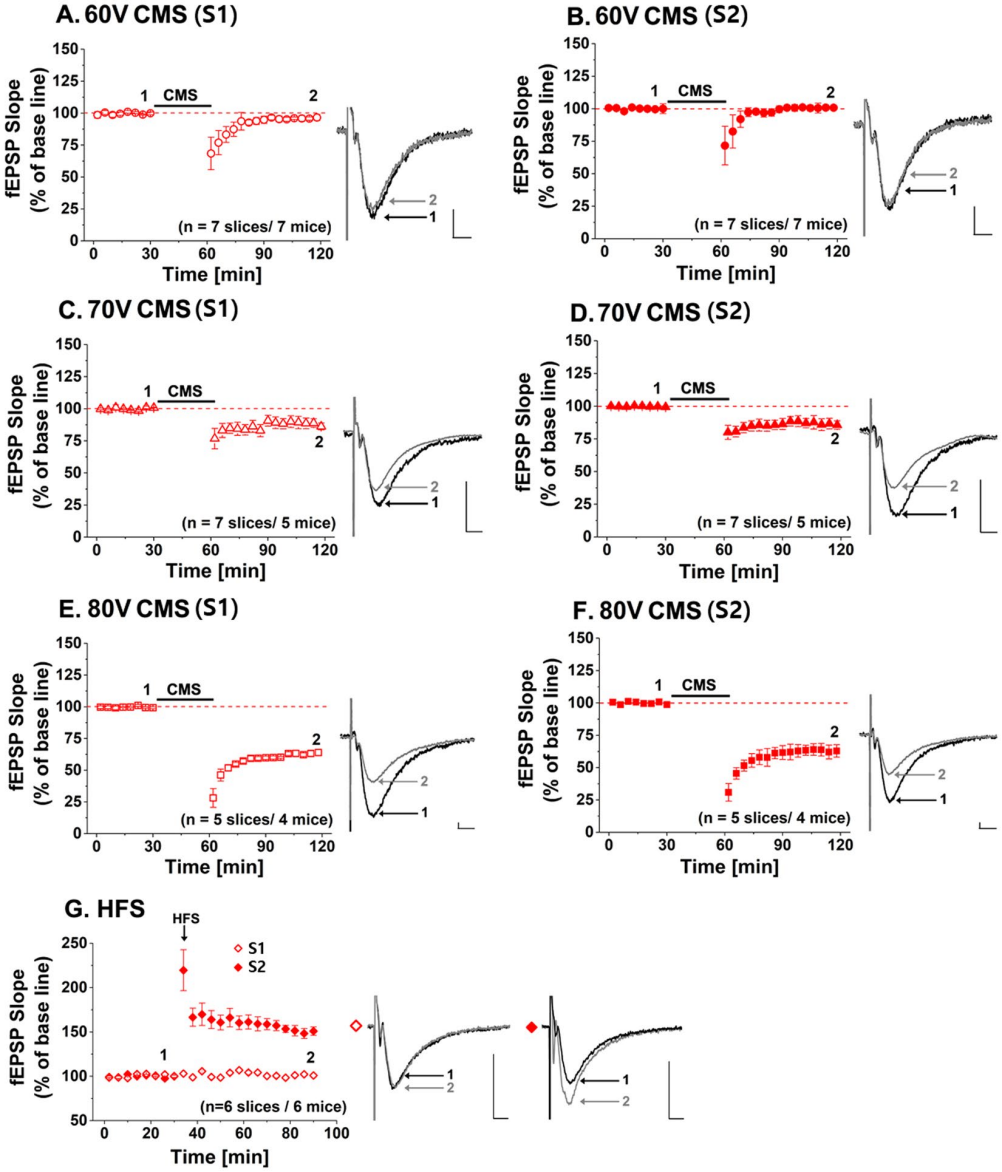
Synaptic responses before and after contact-mode magnetic stimulation (CMS) depending on voltage amplitudes of 60 V, 70 V and 80 V, and before and after high frequency stimulation (HFS): Each point in field excitatory synaptic potential (fEPSP) slope corresponds to the mean ± SEM, and is normalized by its baseline value (100%). Normalized fEPSP slopes are shown in each figure with sample traces. The black and grey traces in the insets depict the fEPSP before (1) and after (2) the stimulation, respectively. (**A**,**B**) In CMS, 60 V CMS produces little difference in the fEPSP slope than the baseline. (**C**,**D**) The fEPSP slopes after 70 V CMS decrease to 86 ± 3% and 84 ± 3% by synaptic input S1 and S2, respectively. (**E**,**F**) 80 V CMS seem to significantly reduce the fEPSP slope to 63 ± 2% and 62 ± 4% by synaptic input S1 and S2, respectively. (**G**) Normalized fEPSP slopes are shown to be increased to 149 ± 5% by synaptic input S2 after high-frequency stimulation. The black arrow indicates the time point of HFS (tetanic stimulation) application.

All field excitatory postsynaptic potential (fEPSP) slopes measured over time were displayed as the mean ± SEM in each of 7 experimental groups as shown in Fig. 2. The synaptic responses before and after the CMS are shown in Figs 2A–F. The representative recorded traces are displayed with respect to 60 V, 70 V and 80 V voltage levels. These voltage levels correspond to 1.9 A, 2.2 A, and 2.5 A currents flowing through the coil. These currents were calculated based on a 31 Ω coil resistance. In our previous study^1^, we expect that a current of 1.7 A would be required to modulate neural activities. Therefore, we varied the current level from 1.9 A (60 V) to 2.5 A (80 V). The fEPSPs were evoked by synaptic input in Schaffer collaterals pathway (S2) and CA1/Subiculum (S1), and recorded at CA1 stratum radiatum. The fEPSP slope after 60 V CMS showed minor changes compared to the baseline by S2 (Fig. 2B, 100 ± 1%, n = 7, t(6) = −1.968, p = 0.09), while the fEPSP slope was 5% lower than the baseline by S1 (Fig. 2A, 95 ± 1%, n = 7, Student’s paired t-test, t(6) = 13.895, ***p < 0.001). With 70 V CMS, the fEPSP slopes declined by 14% and 16% by S1 (Fig. 2C, 86 ± 3%, n = 7, t(6) = 17.392, ***p < 0.001) and S2 (Fig. 2D, 84 ± 3%, n = 7, t(6) = 40.221, ***p < 0.001), respectively. When 80 V CMS was applied, significant fEPSP slopes reductions were observed to be 37% by S1 (Fig. 2E, 63 ± 2%, n = 5, t(6) = 49.678, ***p < 0.001) and 38% by S2 (Fig. 2F, 62 ± 4%, n = 5, t(6) = 8.543, ***p < 0.001). As the intensity of magnetic stimulation increased, the amount of synaptic responses became larger. The long-term depression (LTD)-like effect was observed in CA1 by synaptic input S1 and S2 after CMS of the whole area of the hippocampal slices.

**Figure 3:**
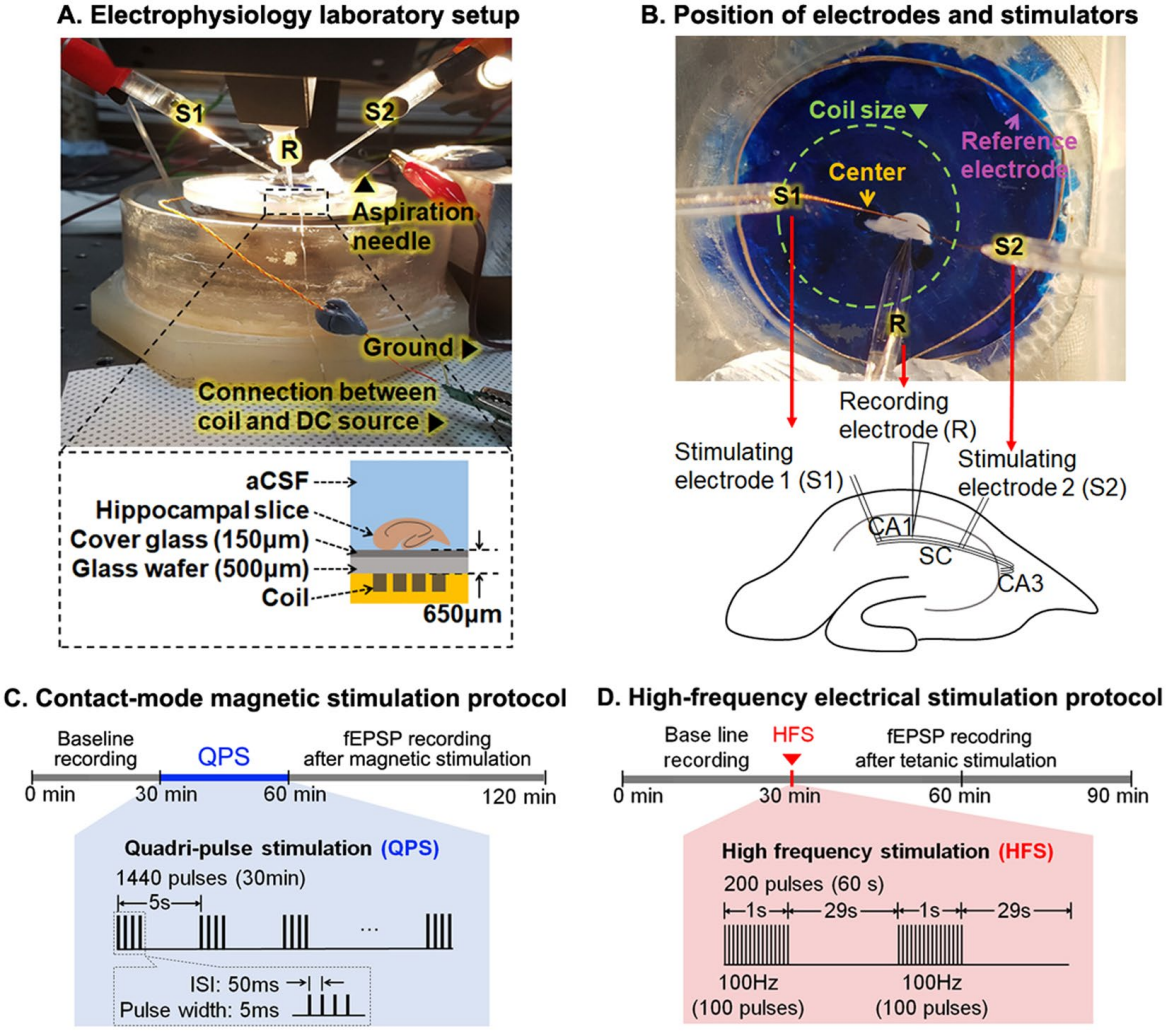
Electrophysiological recording setup and experimental protocols: (**A**) Photograph of the recording chamber and schematic drawing of its cross-sectional side view (lower panel). Hippocampal slice is about 650 μm away from the coil. (**B**) Microscopic image of the recording chamber from the top view. The image shows the location of the coil, hippocampal slice, and a reference electrode. S and R represent the stimulating and recording electrode, respectively. The recording electrode (R) is located in CA1. The stimulating electrodes S1 and S2 are located CA1/Subiculum and Schaffer collateral pathways. (**C**) Contact-mode magnetic stimulation (CMS) protocol for 120 min. QPS-patterned CMS is applied for 30 min after baseline recording. QPS-patterned CMS is composed of the pulse width of 5 ms, inter-stimulus interval (ISI) of 50 ms, inter-train interval (ITI) of 5 s, and a total number of the stimulus of 1440. (**D**) LTP protocol along with the timeline. High-frequency stimulation (HFS) pattern of 100 Hz for 1 s is applied twice with the interval of 30 s to the hippocampal slice.

We also applied high-frequency electrical stimulation to Schaffer collateral of hippocampal slices in order to confirm the slice conditions as well as the electrophysiological setup conditions. The stimulation results were compared with the results by the CMS. High-frequency stimulation (HFS) is well known for inducing LTP effects in the hippocampal slice^28,29^ and is also called ‘tetanic stimulation’. The HFS protocol is shown in Fig. 3D. A stimulation train of 100 pulses at 100 Hz repetition rate was applied to Schaffer collateral twice with a 30 s inter-train interval. Figure 3G shows synaptic responses before and after HFS. The fEPSP slope increased by 49% compared to the baseline recording (149 ± 5%, n = 6, Student’s paired t-test, t(6) = −54.821, ***p < 0.001), as shown Fig. 2G. As expected, the LTP effect was maintained for 1 hour after HFS, which was in good agreement with previous results by others^33,34^. This finding validated our electrophysiological recording setup.

**Figure 4:**
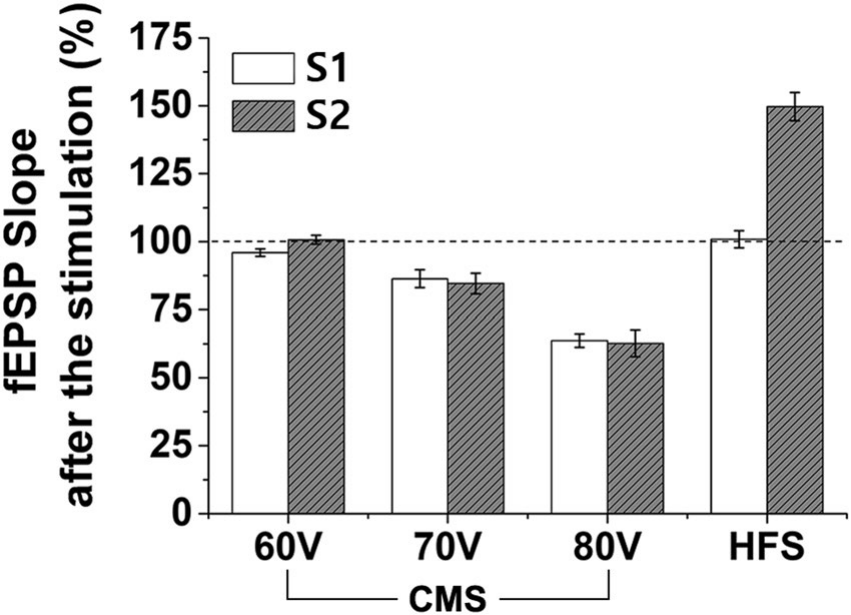
Changes of normalized fEPSP slope after the CMS and HFS stimulation normalized by baseline in response to synaptic input S1 (60 V: 95.9 ± 1.3%, 70 V: 86.3 ± 3.3%, 80 V: 63.6 ± 2.4%, and HFS: 100.8 ± 3.1%) and S2 (60 V: 100.7 ± 1.5%, 70 V: 84.6 ± 3.7%, 80 V: 62.6 ± 4.9%, and HFS: 149.7 ± 5.2%).

Figure 4 shows the summary of the recorded fEPSP data at different stimulation conditions, which are normalized to those of the baseline recording. In CMS, the fEPSP slopes decreased as the stimulating voltage amplitude increased. In comparison, the LTP effect was shown in the Schaffer collateral-commissural pathway in HFS.

### Temperature rise during contact-mode magnetic stimulation

**Figure 5:**
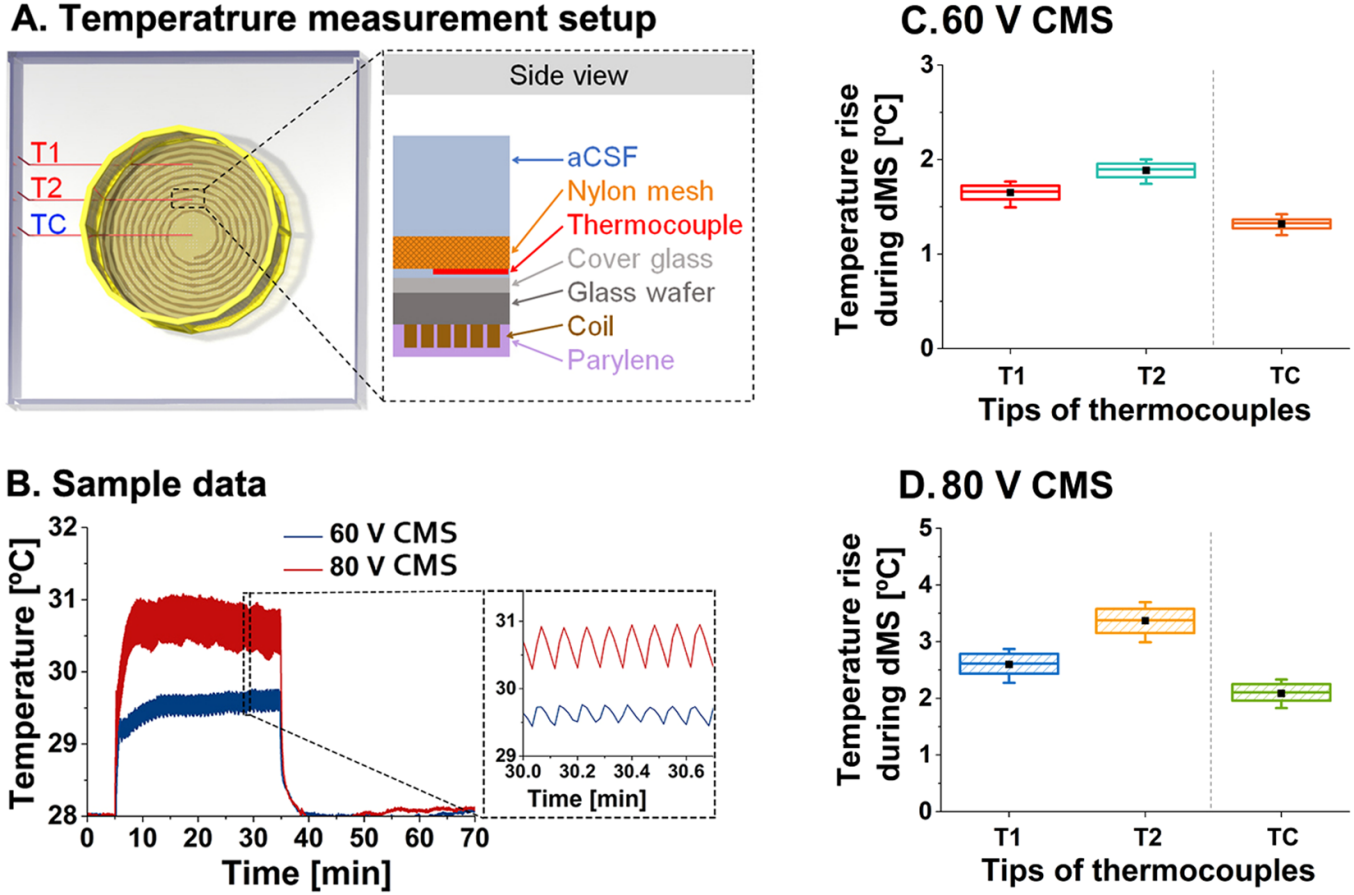
Experimental setup and results of temperature measurement around the coil: (**A**) Three-dimensional schematic top view of the experiment setup in the left illustration. T1 and T2 are located over the wires of the coil while TC is located at the center of the coil. The more details from the side view are described in the right. The tips of the thermocouples were located on the cover glass and physically fixed by a nylon mesh. (**B**) Sample traces of temperature changes caused by 60 V (blue) and 80 V (red) CMS. The magnified temperature measurement during 60 V and 80 V CMS is displayed in the right dashed square box. (**C**,**D**) The average steady-state temperatures, measured from 5 trials per each tip of thermocouples by 60 V and 80 V CMS (data set for 29–34 min shown in Fig. 5B), are drawn in box graph (square box in a 25–75% range; —: maximum and minimum values; filled black square: mean). The range of square box was 25–75% of the temperature change due to the repeated stimulation train of QPS-patterned CMS every five seconds. 60 V CMS increased the temperature by 1.64 ± 0.08 °C, 1.88 ± 0.07 °C and 1.31 ± 0.05 °C in T1, T2, and TC positions, respectively. 80 V CMS increased the temperature by 2.59 ± 0.18 °C, 3.36 ± 0.22 °C and 2.08 ± 0.15 °C in T1, T2, and TC positions, respectively.

The coil in this study was small enough to be implanted in the brain for potential human applications. Unfortunately, the small coil had relatively high resistance, and there was a risk of high heat generation. Hence, we measured the temperature to investigate the heat generated around the coil during QPS-patterned CMS. Three T-type thermocouples were used to measure temperatures around the coil. Two welded tips were located in T1 and T2 as shown in Fig. 5A. The hippocampal slice was placed over these tips in the electrophysiological recording setup. The third thermocouple (TC) tip was located at the center of the coil, where no conducting coil traces were present.

An example where the temperature was measured for 70 min is shown in Fig. 5B. The temperature reached the steady-state after approximately 15 min after the initiation of CMS, and CMS was applied for the next 30 min. After CMS, the temperature during heat dispersion was subsequently measured for 30 min. In CMS, the temperature fluctuated slightly with a 5 s period, which corresponded to the inter-stimulus interval of the QPS pattern (a magnified view is shown in the inset of Fig. 5B). The temperature returned to the base temperature within 5 min after CMS was terminated. The base temperature in all experiments was between 28 °C and 29 °C. Figures 5C and 5D show the temperature changes by 60 V and 80 V stimulation voltage, respectively. In 60 V CMS, the temperature rose by 1.64 ± 0.08 °C (T1), 1.88 ± 0.07 °C (T2), and 1.31 ± 0.05 °C (TC) in each position. In 80 V CMS, the temperature rises were slightly larger, taking values of 2.59 ± 0.18 °C at T1, 3.36 ± 0.22 °C at T2 and 2.08 ± 0.15 °C at TC. Overall, the temperature changes during 60 V and 80 V CMS were less than 2 °C and 3.5 °C, respectively. This, in turn, resulted in maximum temperatures of 30.8 °C and 32.3 °C during 60 V and 80 V CMS relative to the base temperature of 29 °C.

## Discussion

Implantable magnetic stimulation was recently suggested and studied for its feasibility in *in vitro*^18^ and *in vivo*^20^ conditions, suppression of subthalamic nucleus activity^19^, eliciting neural activities of cortical pyramidal neurons^22^, and development of penetrating-type stimulating microcoils^2^. Although neuromodulation effects using coils developed by each research team have been extensively studied, there has been little research on the heat generated around the coil during magnetic stimulation. Here, we evaluated the stimulation effects in hippocampal slices through CMS using fabricated planar coils and subsequently measured the temperature rise during CMS.

First of all, we found that more long-lasting synaptic depression was induced as the stimulation intensity of CMS increased. LTD is a type of synaptic plasticity and is involved in long-lasting synaptic inhibition^35^, contrary to the LTP effect evoked by HFS. In neuroscience, the LTP effect indicates a persistent strengthening of synapses, which produces a long-lasting enhancement in signal transmission between neuronal terminals^36^. TMS is known to induce persistent changes in hippocampal synaptic plasticity in *in vivo*^27,37^ and *in vitro*^28,29^. TMS-based *in vivo* studies have primarily focused on finding methods to treat neurodegenerative^27,38,39^ or psychiatric diseases^40^. *In vitro* TMS experiments investigated the effects used to control the signal pathway of specific synaptic proteins^41^, as well as changes in the functional and structural plasticity of postsynapses in organotypic cultured slices^30,31,42^ or acute slices^28,29,43^. Interestingly, results from prior studies indicate that neuronal or synaptic responses tend to depend on frequency. Excitatory or LTP-like effects were induced by high-frequency TMS^27–30^, while inhibitory or LTD-like effects were evoked by low-frequency TMS^41,43^ in the hippocampus, although not all of the studies show the same results. The QPS-patterned CMS used in the present study consisted of an inter-train interval of 0.2 Hz and inter-stimulus interval of 20 Hz. Although the used QPS pattern contained both high- and low-frequency components, our QPS-patterned CMS results show that the used inter-train interval was more likely to induce long-lasting synaptic depression.

To see whether this synaptic depression may have been caused by cell death upon magnetic stimulation, we performed priming magnetic stimulation (PMS)^44^. This protocol was explained in Fig. S1 of the supplementary information. The LTP effect was not observed when HFS was given immediately after the end of CMS application, but it was observed when HFS was given at times later than 90 min after the end of CMS, as shown in Fig. S2. These observations indicate that CMS affected synaptic activity only for a certain period of time without cell damages.

After electrophysiological measurements, the temperature surrounding the coil was measured during CMS to investigate the thermal effects of CMS. The highest measured temperatures were 30.8 °C and 32.3 °C for 60 V and 80 V voltages, respectively. In the electrophysiological experimental setups used by others, the aCSF temperature was set to be between 28 °C and 34 °C for fEPSP recording in the hippocampal slice^45–47^ and perirhinal cortex^48^. Theta-like activity was also observed from the hippocampal slice within a temperature range of 28 °C to 39 °C. In particular, the most active responses in hippocampal slices were observed at temperatures between 33 °C and 37 °C^49^. In light of these results, the increased temperature of 32.3 °C caused by 80 V CMS is preferably within the temperature range at which synaptic responses increased, indicating that the depression of synaptic potentials was not mediated by the heat generated from the coil. In previous studies, it is generally known that the fEPSP slope increases when the temperature rises^50^. Our experiments showed that the fEPSP slope was suppressed even though the temperature rose during magnetic stimulation. Therefore, it is concluded that the synaptic depression was caused by factors other than the temperature, namely, by the presence of magnetic stimulation.

Previously, implantable magnetic stimulation has been speculated to cause cell damages by heat from the coil. Thus, the micro-coils were located apart from the brain slices or cortical surface, not in contact with the tissues, in previous studies on implantable magnetic stimulation by others^18–20^. However, we investigated the thermal feasibility of implantable coils used for magnetic stimulation, by showing the amount of temperature rises due to magnetic stimulation in the tissue in contact with the coil, for the first time. Therefore, the experimental result of temperature measurement during CMS is expected to provide information for the potential use of the CMS coil as an implantable device.

Assuming such a coil used in the present study could be implanted on the brain surface, it is expected that the temperature would rise to a maximum of 38 to 39 °C according to FEM simulation results as shown in Fig. S3 of the supplementary information. As the coil would be surrounded by cerebrospinal fluid, and heat transfer through conduction and convection becomes more active in the real brain, the temperature in *in vivo* conditions would be lower than that measured in *in vitro* conditions. Also, the simulation results indicate that the temperature increase on the gray matter surface depends on the thickness of the coil’s insulation layer. In addition, it is shown that polymeric insulation materials would effectively prevent rapid temperature rises on the gray matter surface because they have larger heat capacity and lower thermal conductivity than those of the glass that was used in the present *in vitro* experiments. Fekete and colleagues recently found that the brain tissue was not damaged by increased temperature up to 39 °C^24^. In addition, no changes in blood-brain barrier permeability or cell deaths in the brain were observed due to whole-body heating up to 41 °C^25^. In light of these studies, the planar coil used in the current study has the potential to sufficiently stimulate or modulate neural tissues as an implantable stimulation system without thermal damage. Although we saw the potential of the planar coil-based magnetic stimulation as an implantable stimulation device, more investigations are needed to clarify the safety issue about local temperature increases around the coils by considering brain temperature fluctuations by a variety of activities in *in vivo* animal models^51–53^. This planar-type coil can also stimulate a much more localized cortical area than TMS, especially when implanted through a minimally invasive procedure such as that used for the implantation of cochlear implants on the thinned skull. Such planar coil-based CMS devices may be applied to treat Parkinson’s disease^54,55^, Alzheimer’s disease^56,57^ or schizophrenia^58,59^.

In summary, The QPS-patterned CMS induced a weakening of synaptic activities and directly elicited long-lasting synaptic depression at CA1 synapses in the hippocampal slice. These results were obtained regardless of the heat generated from the coil during CMS. The temperature rise did not significantly modulate synaptic activities in the hippocampal slice. In the future, the molecular and cellular mechanisms of CMS need to be further investigated using methods such as a western blotting^60^ or immunohistochemistry^61,62^ to analyze the variation of crucial proteins for synaptic LTD; calcium imaging^63^ to confirm calcium signaling in network-level; Terminal deoxynucleotidyl transferase dUTP nick-end labeling (TUNEL) to detect possible cell depth^64,65^; patch-clamp recording using various receptor blockers^22,29,31^; input-output curve for analyzing basal synaptic transmission^44^; paired-pulse ratio to measure the short-term plasticity in the presynaptic terminal. Also, more experimental studies using various combinations of CMS parameters such as pulse width, ISI, ITI and duration of stimulation are desired.

## Methods

### Coil fabrication

The coil fabrication process (Fig. 1A) begins with the cleaning of a borosilicate glass wafer (500 μm thick) using the standard cleaning process. After cleaning, a 100 nm titanium (Ti) layer was deposited on the glass substrate. This layer functioned as an adhesion layer between glass and copper (Cu). A 600 nm Cu layer was deposited on the Ti layer, as a seed layer for electroplating. A 35–38 μm thick photoresist (AZ 40 XT-11D, AZ Electronic Materials, Darmstadt, Germany) was patterned using a photolithography technique. Before electroplating, a descum process was carried out to remove photoresist residues where the coil would be developed using a microwave asher. A 30 μm thick copper layer was electroplated (SW-PM2-R2Q1, Sungwon forming, Korea) to form the conductive lines of the coil. After electroplating, the photoresist mold was removed using a remover (TechniStrip® NI555, AZ Electronic Materials, Darmstadt, Germany) and the seed layers were etched by Cu and Ti etchants (Cu ETCH 49-1 and Ti ETCH TFT, Transene Inc., Danvers, MA, USA). Before the dicing process was used to separate individual coils, a thin photoresist layer (AZ1512, AZ Electronic Materials, London, UK) was coated on the conductive lines to keep micro residues from getting into the space between the coil lines. Each coil was cut into a hexagonal shape using an automatic dicing saw (DAD3350 (DISCO), Giorgio technology, Mesa, AZ, USA). After dicing, the protective photoresist layer was removed. Thin conductive wires with 240 μm diameter were connected by soldering from each end of the coil to the customized PCB board. These coils were finally passivated with a 15 μm Parylene-C layer using LPCVD (PDS 2010, Specialty Coating Systems, Indianapolis, IN, USA). A microscopic image of a fabricated coil is shown in Fig. 1B. After passivation of the coil, we measured the coil impedance using a precision impedance analyzer (4294A, Agilent Technologies, Santa Clara, CA, USA). Electrical measurements were used to confirm that the impedance and inductance values of the coil were adequate for use in CMS.

### Animal and brain slice preparation

All experiments were performed based on permission from the committee on animal research and ethics at Chonnam National University Medical School (CNUIACUC-H-2018-1) and Gwangju Institute of Science and Technology (No. GIST-2016-09). Hippocampal slices were prepared from seven- to eight-week-old male C57BL/6 mice. They were kept with the standard 12 h light/dark cycle, with light commencing at 8 a.m. All data were acquired from the hippocampal slices from 15 animals, and each of the slices was stimulated by one of the used protocols. All mice were decapitated immediately after cervical dislocation between 9 a.m. and 11 a.m. The hippocampi were rapidly dissected from each hemisphere and soaked in iced oxygenated artificial cerebrospinal fluid (aCSF). The aCSF solution was freshly made before each experiment by dissolving the following composition (7.25 g NaCl, 2.18 g NaHCO_3_, 1.8 g glucose, 0.25 g MgSO_4_, 0.22 g KCl, and 0.29 g CaCl_2_·H_2_O; equilibrated with 95% O_2_−5% CO_2_)^66^ in 1 L of deionized water. The isolated hippocampi were transversely sectioned at 350–400 μm using a McIlwain tissue chopper (Mickle Laboratory Engineering Co. Ltd., Gomshall, UK), and then incubated in the oxygenated aCSF solution at room temperature (24–27 °C) for at least two hours before electrophysiological recordings.

### Electrophysiological recording and stimulation conditions

Recording pipettes of borosilicate glass (Harvard Apparatus, Holliston, MA, USA) were pulled from a glass pipette puller (P-1000, Sutter Instrument, Novato, CA, USA). All fEPSP measurements from the hippocampal slices were recorded by the glass pipette of about 2 MΩ impedance filled with 3 M NaCl. An Axopatch 200A amplifier (Axon Instruments, Foster City, CA, USA) was used for voltage clamp recordings. The evoked fEPSP signals were low-pass filtered at 5 kHz using a 4-pole Bessel filter and acquired at a 10 kHz sampling rate using a data acquisition board (NI PCI-6251, National Instruments, Austin, TX, USA) linked to WinLTP software (Anderson and Collingridge, 2007; http://www.winltp.com).

The hippocampal slice was transferred to the recording chamber, under which the fabricated coil attached to a cover glass was placed, as shown in Fig. 3A. Magnetic stimulation through the fabricated coil was applied to the hippocampal slice in a direction such that axons in the CA1 region were parallel with the conducting lines of the coil, as shown in Fig. 3B. It is already known that the parallel orientation of hippocampal slices with respect to the coil traces exhibits lower thresholds than the perpendicular orientation under magnetic stimulation^19^. Therefore, we used this orientation to induce neural activities using as low electrical energy as possible. The temperature in the recording chamber was maintained at 28–30 °C by perfused aCSF solution at a rate of 3 ml/min. The temperature controller was located outside the recording chamber and maintained the temperature of aCSF flowing to the chamber constantly.

We measured the hippocampal synaptic function by recording the fEPSP in the CA1 stratum radiatum (by the recording electrode ‘R’) in response to electrical stimulation inputs to the Schaffer collateral (by the stimulating electrode ‘S2’) and CA1/Subiculum (by the stimulating electrode ‘S1’), as shown in Fig. 3B. The initial amplitude of the recorded fEPSP signal was adjusted to about 30% of the maximum fEPSP slope. Typical maximum fEPSP slopes range from −0.7 to −1.5 V/sec^67^. 100 μs electrical pulses were used to evoke a synaptic response and were delivered at 15 s intervals through twisted bipolar Ni-Cr alloy electrodes. The electrodes were alternately placed at the CA1/Subiculum and Schaffer collateral, as shown in Fig. 3B (denoted by ‘S1’ and ‘S2’). The fEPSP slope was averaged over four potential signals and normalized with respect to the mean fEPSP slope during baseline recording, i.e., before electrical or magnetic stimulation.

In this study, we applied electrical and magnetic stimulation to hippocampal slices. Figure 3C shows the QPS-patterned CMS protocol. The QPS consisted of pulse trains with four 5 ms pulses, each separated by an inter-stimulus interval (ISI) of 50 ms. After recording a baseline for 30 min, CMS was applied for 30 min, followed by the measurement of the fEPSPs for 1 hour. Magnetic stimulation with 60 V, 70 V, and 80 V pulses were applied to the entire hippocampal slice located above the coil. This configuration allowed the effects of magnetic stimulation to be observed in the CA1 stratum radiatum by both the stimulating electrodes S1 and S2, both of which were placed above the coil turns. For comparison to CMS, HFS was used as electrical stimulation protocol, which consisted of two 100 pulses, each with a 1 s duration and a 30 s interval between each train, as shown in Fig. 3D. Electrical stimulation was delivered to the Schaffer collateral to induce the LTP effect after recording a baseline for 30 min. The stimulation intensity was the same as that used during baseline recording.

To determine the appropriate magnetic stimulation protocols, we tried many rTMS patterns^68^, such as regular high-frequency rTMS, continuous or intermittent theta burst stimulation (cTBS or iTBS), and quadripulse stimulation (QPS) (data or figures are not shown). Among them, the QPS pattern was chosen as the proper stimulation pattern for our experimental setup. Using QPS, thermal damage to the coil was not considerable compared to other tested stimulation patterns due to the long inter-train interval in the QPS pattern. In our previous study^1^, we found that the inter-stimulus interval (ISI) and inter-train interval (ITI) were the key factors that determine the temperature rise of the coil. Based on the previous study, we chose a QPS pattern consisting of four 5 ms pulses with 50 ms ISI and 5 s ITI. A total of 360 trains were delivered for 30 min during CMS (Fig. 3C). This stimulation pattern was created by programming an AC/DC power supply (AST 751, AMETEK Inc., Berwyn, PA, USA).

### Temperature measurement

The temperature around the coil was measured to estimate the heat delivered to the hippocampal slice during CMS. T-type thermocouples (5TC-TT-T-40–36, Omega Engineering Inc., Norwalk, CT, USA) with welded tip ends were used. The tips were insulated with perfluoroalkoxy (PFA), and the tip end had a small diameter of 0.08 mm. A cover glass was attached to the coil and thermocouples were placed on top of the cover glass. This configuration is similar to the electrophysiological recording setup, as shown in Fig. 5A. The bottom part of a cell strainer (nylon mesh size of 100 μm, Corning, New York, NY, USA) was modified to eliminate space between the mesh and the cover glass. After removing the bottom nylon mesh, a nylon stocking was attached to the bottom frame. Thermocouple tips were inserted between the cover glass and the attached nylon mesh, and the bottom frame of the cell strainer was fixed with glue. Three thermocouples were placed at ~3 mm intervals. One was located at the center of the coil (TC in Fig. 5A) over the location of the slice, and the other two thermocouples were located above the coil traces (T1 and T2 in Fig. 5A). The aCSF solution was heated to 34 °C in a water bath and flowed into a temperature measurement chamber placed on a 31 °C hotplate. The inlet solution lines were also placed on the 31 °C hotplate to prevent heat loss. The inlet and outlet flow rates were 3.0 ml/min and 3.2 ml/min, respectively. The room temperature during the experiment was 22 °C. At the beginning of the first measurement, the temperature was measured for about 30 min to confirm that it was maintained constantly with a fluctuation within ± 0.2 °C. After steady-state was confirmed, CMS voltage pulses (AST 751, AMETEK Inc., Berwyn, PA, US) were applied to the coil. A data acquisition module (NI 9211, 4-Channel, ±80 mV, 24 Bit, 14 S/s, National Instrument, Austin, TX, USA) and LabVIEW were used to collect the temperature data. After applying the QPS-patterned CMS for 30 min, the temperature was measured for an additional 30 min.

### Statistical Analyses

Student’s (one-tailed) t-test was used to compare before-and-after observations on the same subjects after Lilliefors normality test of obtained data. All statistical analyses were performed using Matlab (MATLAB R2013b, Mathworks, Natick, MA, USA). The statistical significance level was set at 0.05 and presented by asterisks (***p < 0.001, *p < 0.05).

### Ethical considerations

All animal care and experiments were approved by the Institutional Animal Care and Use Committee of Chonnam National University Medical School and Gwangju Institute of Science and Technology, and conducted in accordance with relevant guidelines and regulations in Republic of Korea.

## Electronic supplementary material

Supplementary information
