# Supplementary material for: Planar coil-based contact-mode magnetic stimulation: synaptic responses in hippocampal slices and thermal considerations
Source: Sci Rep. 2018 Sep 7;8:13423. doi: 10.1038/s41598-018-31536-w (PMC6128857; doi:10.1038/s41598-018-31536-w)
Supplement: Supplementary file 1 — Supplementary information [file 41598_2018_31536_MOESM1_ESM.docx]

# Supplementary information

# Planar coil-based contact-mode magnetic stimulation: synaptic responses in the hippocampal slices and thermal considerations

H. Park^1^, H. K. Kang^2^, J. Jo^3^, E. Chung^1,4*^ and S. Kim^5*^

^1^School of Mechanical Engineering, Gwangju Institute of Science and Technology (GIST), Gwangju, Republic of Korea;

^2^NeuroMedical Convergence Laboratory, Department of Biomedical Science and Neurology, Chonnam National University Medical School, Gwangju, Republic of Korea;

^3^NeuroMedical Convergence Laboratory, Biomedical Research Institute, Chonnam National University Hospital, Gwangju, Republic of Korea;

^4^Department of Biomedical Science and Engineering, Institute of Integrated Technology (IIT), Gwangju Institute of Science and Technology (GIST), Gwangju, Republic of Korea;

^5^Department of Robotics Engineering, Daegu Gyeongbuk Institute of Science and Technology (DGIST), Daegu, Republic of Korea;

*Correspondence and requests for materials should be addressed to E. C. (email: ogong50@gist.ac.kr) or S. K. (email: [soheekim@dgist.ac.kr](mailto:soheekim@dgist.ac.kr))


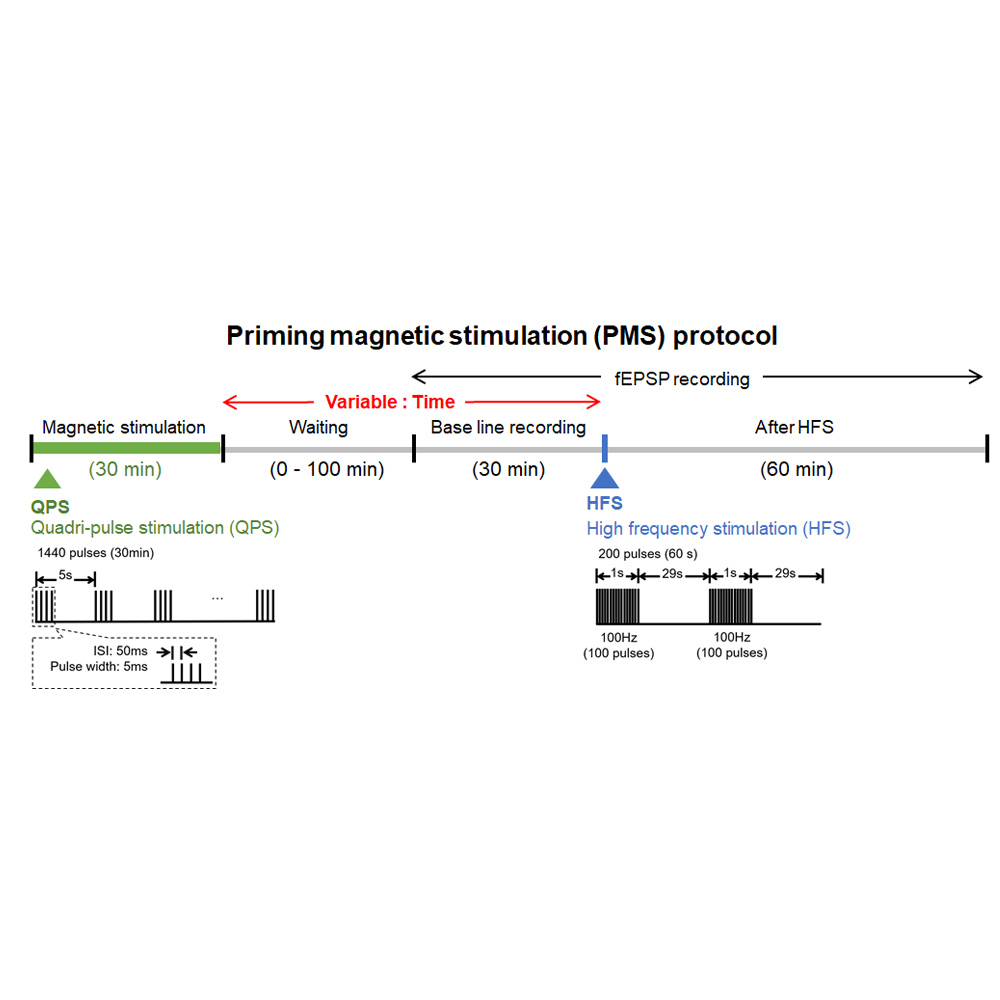


Figure S1. Priming magnetic stimulation protocol with both quadri-pulse stimulation (QPS) and high-frequency stimulation (HFS). The QPS patterned magnetic stimulation was applied before the fEPSP recording. The fEPSP recording started after a certain time ranging from 0 min to 100 min. High frequency electrical stimulation was applied to SC following baseline recording for 30 min.


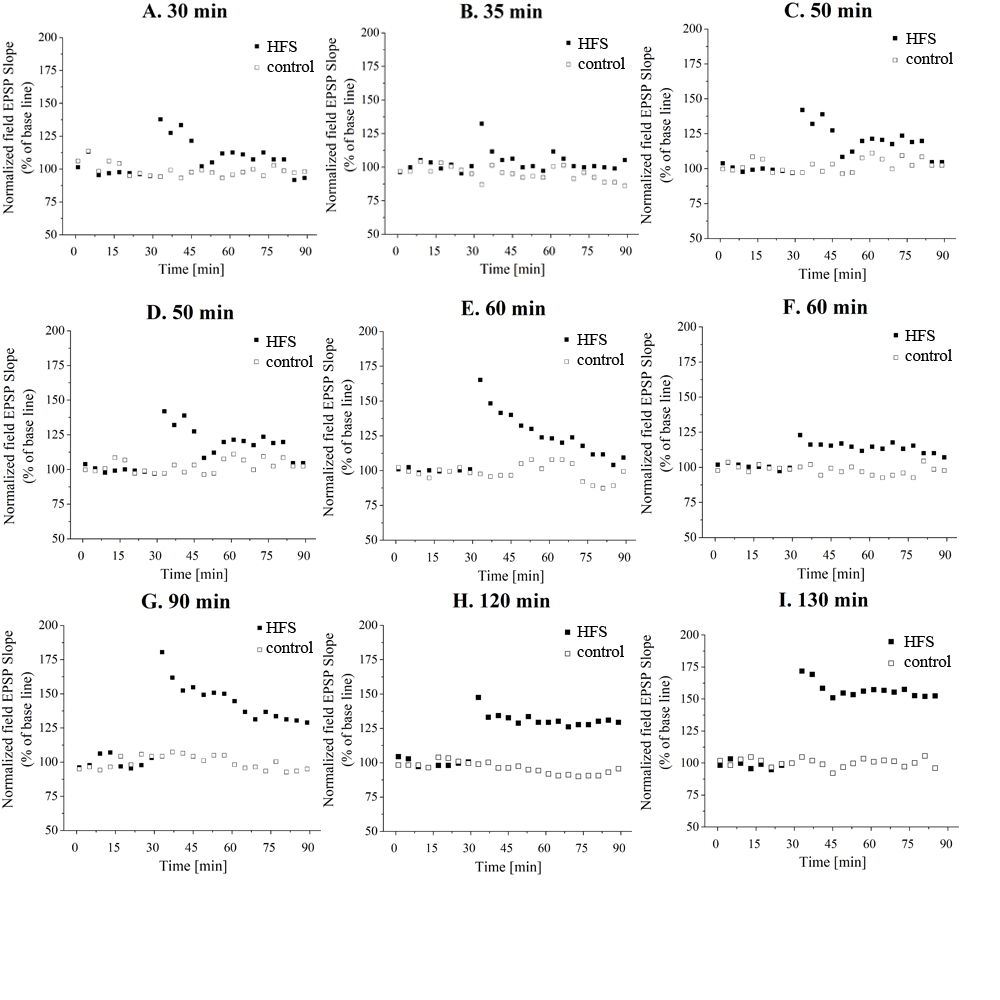


Figure S2. Long-term potentiation responses depending on the time period between the end of CMS and the start of HFS. Long-term potentiation responses were shown when HFS was applied at times later than 90 min after the application of CMS, which indicates that cells were not significantly damaged due to magnetic stimulation.

| **A** | 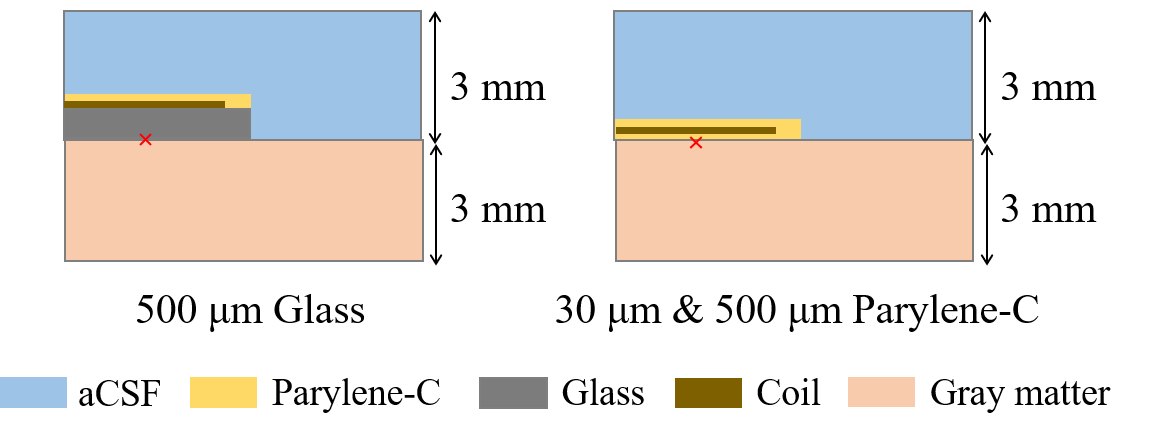 |
| --- | --- |
| **B** | 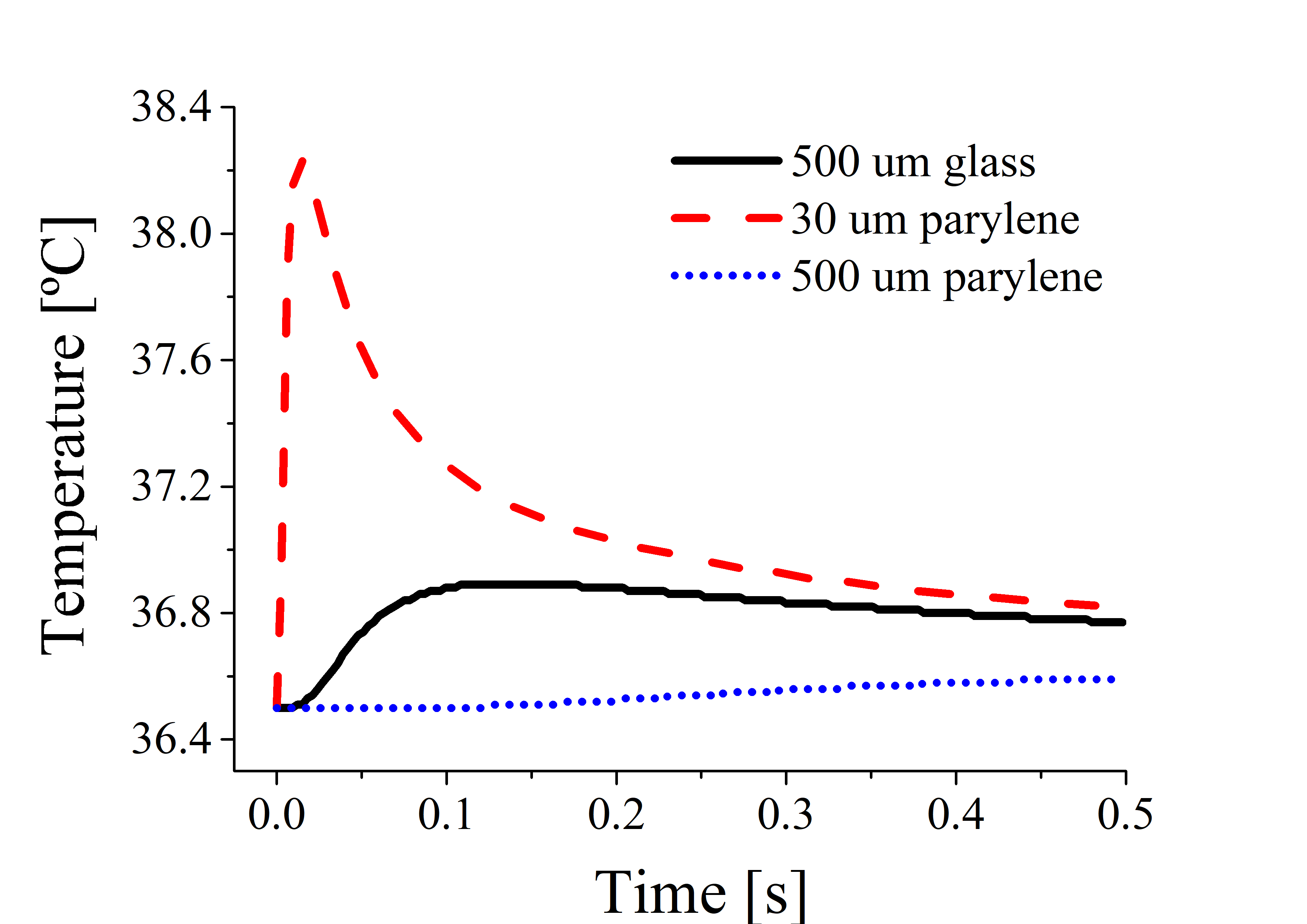 |

Figure S3. FEM simulations to estimate the temperature increase when the coil is implanted on the brain surface. **A**. The axisymmetric model consisted of aCSF, gray matter and planar coil with glass or parylene-C substrate. The employed material and thickness of coil substrates were 500 μm glass, 30 μm and 500 μm parylene-C. The x mark in red indicates the location for data extraction on the gray matter surface. **B**. The simulated temperature increase during magnetic stimulation depending on the material and thickness of insulation layers of the coil.
